# SCRAPT: an iterative algorithm for clustering large 16S rRNA gene data sets

**DOI:** 10.1093/nar/gkad158

**Published:** 2023-03-13

**Authors:** Tu Luan, Harihara Subrahmaniam Muralidharan, Marwan Alshehri, Ipsa Mittra, Mihai Pop

**Affiliations:** Department of Computer Science, University of Maryland, College Park, 20742 MD, USA; Center for Bioinformatics and Computational Biology, University of Maryland, College Park, MD 20742, USA; Department of Computer Science, University of Maryland, College Park, 20742 MD, USA; Center for Bioinformatics and Computational Biology, University of Maryland, College Park, MD 20742, USA; Department of Computer Science, University of Maryland, College Park, 20742 MD, USA; Department of Computer Science, University of Maryland, College Park, 20742 MD, USA; Department of Computer Science, University of Maryland, College Park, 20742 MD, USA; Center for Bioinformatics and Computational Biology, University of Maryland, College Park, MD 20742, USA

## Abstract

16S rRNA gene sequence clustering is an important tool in characterizing the diversity of microbial communities. As 16S rRNA gene data sets are growing in size, existing sequence clustering algorithms increasingly become an analytical bottleneck. Part of this bottleneck is due to the substantial computational cost expended on small clusters and singleton sequences. We propose an iterative sampling-based 16S rRNA gene sequence clustering approach that targets the largest clusters in the data set, allowing users to stop the clustering process when sufficient clusters are available for the specific analysis being targeted. We describe a probabilistic analysis of the iterative clustering process that supports the intuition that the clustering process identifies the larger clusters in the data set first. Using real data sets of 16S rRNA gene sequences, we show that the iterative algorithm, coupled with an adaptive sampling process and a mode-shifting strategy for identifying cluster representatives, substantially speeds up the clustering process while being effective at capturing the large clusters in the data set. The experiments also show that SCRAPT (Sample, Cluster, Recruit, AdaPt and iTerate) is able to produce operational taxonomic units that are less fragmented than popular tools: UCLUST, CD-HIT and DNACLUST. The algorithm is implemented in the open-source package SCRAPT. The source code used to generate the results presented in this paper is available at https://github.com/hsmurali/SCRAPT.

## INTRODUCTION

Sequence clustering is commonly used for a wide spectrum of biological analysis applications (1,2) and particularly in the context of amplicon-based microbial diversity analysis (3,4). Many microbial studies use a survey of conserved marker genes, such as the 16S rRNA gene, as a first step in the analysis of the taxonomic makeup and diversity of a sample.

In the early days of 16S rRNA gene studies, data analyses relied on phylogenetic inference techniques that require finding a multiple alignment of the sequences—an NP-hard optimization problem (5,6). As sequencing technologies advanced, resulting in larger data sets that could not be efficiently analyzed through phylogenetic techniques, the field transitioned to heuristic-based sequence clustering strategies, implemented in popular tools such as wcdEST (7), CD-HIT (8), UCLUST (9), DNACLUST (10), VSEARCH (11) and MOTHUR (12). The clustering algorithms result in operational taxonomic units (OTUs) that can be further used for specific compute-intensive phylogenetic tasks by significantly reducing the complexity of the data sets. Due to the inherent sparsity of microbiome data sets and sequencing errors, singletons and extremely small clusters represent a large fraction of the output of amplicon clustering tools, yet these small clusters are likely to not be used by downstream analyses of the data (13). As data sets continue to grow in size, and clustering algorithms (even heuristic ones) require supralinear run times, we ask: Can the clustering be efficiently performed if we restrict the analysis to just the largest clusters in the data set?

Note that doing so is not a trivial matter as the size of clusters is not known prior to completing the clustering process. We describe here an iterative clustering framework that relies on adaptive sampling to bias the clustering process toward the most abundant clusters in the data set. This approach is implemented in the software package SCRAPT (Sample, Cluster, Recruit, AdaPt and iTerate). We describe a probabilistic analysis of the performance of the iterative clustering process, and show that the implementation of this process in SCRAPT is able to discover the large clusters in a sample in a fraction of the time required by traditional clustering techniques. The iterative approach we describe here enables researchers to trade off run time for the size of clusters that are reliably identified, thus allowing the tuning of the clustering process to the specific analytical needs of individual projects.

Several tools have been described in the literature for performing 16S rRNA gene sequence clustering at scale. CD-HIT (8) is one of the earliest tools developed to perform sequence clustering at scale and many of the subsequent tools largely follow its broad design. CD-HIT processes the set of sequences in decreasing order of length, iteratively comparing each sequence to a set of cluster representatives. A sequence is assigned to a cluster if it aligns to the clusters’ representative with a higher level of similarity than a predefined and fixed threshold. If a sequence does not match any of the pre-existing clusters, it becomes the representative for a new cluster. Tools such as UCLUST (9), USEARCH (9), VSEARCH (11) and DNACLUST (10) adopt a similar strategy. Despite substantial improvements to run times over the original CD-HIT, the run times of these tools are highly dependent on the input similarity threshold and are still stymied by the large data sets currently being generated.

A number of alignment-free approaches have also been developed that achieve further efficiency (14–18). These tools, however, can no longer guarantee a certain sequence similarity within clusters.

For each cluster, the approaches described above use one of the sequences in the input as cluster representative, and the chosen representative remains fixed throughout the execution of the algorithm. In MeShClust (19), the representative gets adjusted as more sequences are considered, using a process called mean shift, in order to ensure that the representative sequence represents the ‘mean’ of the sequences assigned to each cluster. MeShClust also incorporates an intelligent classifier-based strategy to assign unclustered sequences to clusters. In AbundantOTU (20), the cluster centers are not assumed to be sequences from the input, rather are determined through a greedy approach seeded by high-frequency *k*-mers, after which sequences are assigned to clusters by sequence alignment.

The approaches we have mentioned perform a variant of ‘hard’ clustering, in the sense that sequences are assigned to a cluster based on a hard threshold in terms of the similarity between a sequence and the representative sequence for each cluster. In contrast, DADA2 (21) performs a ‘soft’ clustering approach, where the level of similarity between a sequence and the cluster representative is not capped. Instead, clusters are constructed through a divisive partitioning approach that uses as termination criterion an estimate of the likelihood that the input sequences are ‘explained’ by the partitions, under a model of sequencing error and evolution. The centers of the partitions are called amplicon sequence variants (ASVs) to distinguish the results from those that would be obtained through hard clustering. Several studies have shown that DADA2 can more accurately capture biological signal than other approaches, being able to distinguish between closely similar sequences that are indistinguishable by hard clustering algorithms (22,23). DADA2 is the default method for analyzing amplicon sequencing data in QIIME 2 (24).

Finally, we would like to discuss a technique developed to augment closed-reference OTU picking strategies. Closed-reference OTU picking refers simply to using a database of predetermined OTU representative sequences to annotate the data from a sample. Since OTU databases are not comprehensive, closed-reference OTU picking can, thus, miss biologically relevant clusters within a data set. A subsampling approach was proposed in (25) to address this limitation. The algorithm subsamples the sequences that failed to match an OTU in the reference database and then performs *de novo* OTU clustering on subsampled sequences. The OTUs thus identified are then added to the reference database and used in subsequent rounds of closed-reference OTU picking.

### Contributions

In this manuscript, we expand the subsampling strategy described above with a mean-shift approach inspired by MeshClust (19), to develop an efficient iterative *de novo* sequence clustering approach, implemented in the tool SCRAPT. SCRAPT uses adaptive sampling to focus the clustering process on the largest clusters in the data set while retaining computational efficiency and employs a fair center selection step based on sequence multiplicity.

We conduct a theoretical analysis of the clustering process in order to identify the key parameters that determine its efficacy and to provide guarantees about the sizes of clusters that are identified at a particular point in the clustering process. Currently, SCRAPT uses DNACLUST as a clustering kernel. We are using DNACLUST because it implements an efficient ‘baiting’ mode to recruit sequences to predetermined cluster representatives. In future versions of SCRAPT, we will provide support for other clustering kernels and baiting procedures. Using real 16S rRNA gene data sets, we demonstrate the effectiveness of SCRAPT in identifying the large clusters in the data at only a fraction of the computational cost of state-of-the-art OTU clustering tools. Additionally, the run time of SCRAPT is much less affected by the similarity threshold used in clustering, in contrast to DNACLUST, CD-HIT and UCLUST. We also show that the mean-shift approach allows us to match the accuracy of DADA2, thereby providing a practical alternative for large 16S rRNA data sets.

## MATERIALS AND METHODS

We start by briefly presenting the overall structure of the SCRAPT algorithm. The approach starts by selecting a subsample of the data set being clustered and performs clustering on this subsample. The representative sequence of each non-singleton cluster from the subsample is then used as a ‘bait’ to retrieve the other sequences in the full data set that belong to the corresponding cluster. The process repeats iteratively until a user-defined stopping point. At each iteration, SCRAPT uses a mode-shifting strategy to adjust the representative sequences of clusters in order to ensure that these sequences approximate the parent sequence from which the cluster members ‘evolved’ due to short-term evolution and sequencing errors. The SCRAPT algorithm takes as input a set of deduplicated sequences together with their multiplicities, a sequence similarity threshold that defines the radius of the clusters and parameters that define the sampling rate, as will be described in more detail below. We use SeqKit(26) to perform the deduplication and subsampling steps of SCRAPT.

### Naive sampling

We begin by describing and analyzing a naive version of the iterative algorithm. In each iteration, we sample uniformly a small portion (e.g. 0.1%) of the unclustered sequences. The sampled sequences are then clustered with DNACLUST and the non-singleton clusters (clusters containing two or more sequences) are considered for further analysis. The likelihood that two sequences from a taxonomic unit occur within a given sample, allowing the algorithm to ‘detect’ this taxonomic unit, depends on the abundance of the taxonomic unit in the entire data set, with larger clusters being more likely to be identified by any given subsample. As a result, the sampling process biases the clustering toward identifying the most abundant clusters in the sample. Note that sequences that do not cluster within a subsample are not necessarily singletons in the entire data set, and therefore they cannot be eliminated from further consideration and are returned into the pool of unclustered sequences to be processed in subsequent iterations.

The representative sequences for the clusters formed from the subsample are used as ‘baits’ to recruit other sequences from the full data set to the corresponding clusters. We perform this step using the ‘recruitment’ mode of DNACLUST, at a minimum sequence similarity threshold as specified by the user. The sequence similarity defined in SCRAPT is the same as the sequence similarity defined by DNACLUST, as we use DNACLUST for clustering and ‘baiting’. Sequence similarity in DNACLUST is given by $\text{similarity} = 1 - {\text{edit distance}}/{\text{length of the shorter sequence}}$. We run DNACLUST with its default mode where gaps are allowed at the end of a sequence but not at the beginning. During this baiting process, we also adjust the cluster representative using a mode-shifting procedure that will be described in more detail below.

At the end of each iteration, we report the clusters found and remove the clustered sequences from the set of unclustered sequences. The algorithm proceeds either for a user-defined number of iterations or until the number of clusters with a given size identified in each iteration converges. We refer to this process as naive because the sampling rate is held constant from one iteration to another.

### Analysis of the naive sampling

Here, we provide a probabilistic analysis of the naive sampling-based algorithm described in the prior section. The following notations apply: the total number of unclustered sequences at the start of any iteration—*N*; the number of sequences sampled in an iteration—*n*; the number of sequences in cluster *c*_*k*_—*ρ*_*k*_. The sampling rate *α* is given by ${n}/{N}$. A cluster of size *ρ*_*k*_ is identified in a round if there are at least two sequences from the cluster *c*_*k*_ in that sample. Thus, the probability that the cluster *c*_*k*_ is recovered in an iteration that sampled *n* sequences is given by


$$\begin{equation*} p_k = \sum _{i=2}^{{\rm min}(n, \rho _k)} \frac{{N-\rho _k \atopwithdelims ()n-i}{\rho _k \atopwithdelims ()i}}{{N \atopwithdelims ()n}}. \end{equation*}$$

**Lemma 1:** Given *N* sequences and a sample obtained by uniformly sampling *n* sequences, the bound on the probability of missing a cluster *c*_*k*_ of size ρ_*k*_ is given by


$$\begin{equation*} \bar{p}_k = \frac{{N-\rho _k \atopwithdelims ()n-1}\cdot \rho _k}{{N \atopwithdelims ()n}} + \frac{{N-\rho _k \atopwithdelims ()n}}{{N \atopwithdelims ()n}} \le {\rm e}^{-{\rho _k n}/{N}}\left(1 + \frac{\rho _k n}{N}{\rm e}^{{\rho _k}/{N}}\right). \end{equation*}$$

**Figure 1.**
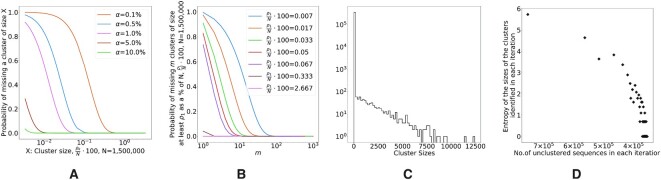
(**A**) Probability of missing a cluster for different values of $({\rho _k}/{N})\times 100$ at different sampling rates $\alpha = ({n}/{N})\times 100,\; N=981\,080$. (**B**) Probability of missing *m* clusters of size at least *ρ*_1_ specified percentage of the total sample size [$({\rho _1}/{N})\times 100$] at a sampling rate of $\alpha =0.1\%,\; N=981\,080$. (**C**) Distribution of cluster sizes. (**D**) Entropy of the cluster sizes discovered in each iteration versus the number of unclustered sequences at the end of each iteration.

Figure 1A plots the probability of missing a cluster of size *ρ*_*k*_ relative to the size of the database for different sampling rates.

Next, we extend Lemma 1 to calculate the probability that the naive sampling strategy would miss *m* clusters $[c_1, c_2, \ldots, c_m]$ of sizes at least $[\rho_1, \rho_2, \ldots, \rho_m]$ in a given iteration.

**Lemma 2:** Given *N* sequences in the database with *n* sequences in the sample, the probability $\bar{p}_k^m$ of missing *m* clusters of size $k=[\rho_1, \rho_2, \ldots, \rho_m]$ such that $\rho_1 \le \rho_2 \le \cdots \le \rho_m$ is given by


$$\begin{eqnarray*} \bar{p}_k^m = \sum _{i=0}^{m} B_{m,i}\times \frac{{N-\sum _{j=1}^{m}\rho _j \atopwithdelims ()n-i}}{{N \atopwithdelims ()n}} \le {\rm e}^{-{m\rho _1 n}/{N}}\left(1+\frac{\rho _1 n}{N}{\rm e}^{{m\rho _1}/{N}}\right)^m ,\\ \text{where }\; B_{i,j} = \left\lbrace \begin{array}{@{}l@{\quad }l@{}}0, &i = 0,\\ B_{i-1,j-1}k_i + B_{i-1,j}, & \text{otherwise}.\\ \end{array}\right. \end{eqnarray*}$$

In Figure 1B, we plot the probability of missing *m* clusters for different cluster sizes. We describe the proofs of the lemmas in Sections 1.1 and 1.2 of Supplementary Data.

The two lemmas described above establish the relationship between the sampling rate *α*, the cluster density *ρ*, the number of sequences in the database and the probability of missing 1 and *m* clusters of size at least *ρ*, respectively. However, one thing that the lemmas do not directly convey is how the distribution of clusters changes in each iteration, a factor that determines whether enough representatives from the clusters of interest are sampled in each round. Since the distribution of cluster sizes is not known in advance, we exemplify the behavior of the algorithm using a set of simulations. In order to mimic a realistic data set, we assumed that 20% of the data set comprises singleton clusters and assumed a probability distribution for the remaining non-singleton cluster sizes. In each iteration, we sample 0.1% of the sequences from the set of unclustered sequences uniformly at random and identify clusters based on flipping a biased coin with the probability calculation shown in Lemma 1 for each cluster remaining in the database. In Figure 1C, we show the distribution of initial cluster sizes sampled from a geometric distribution. In Figure 1D, we show the relationship between the entropy of the cluster sizes discovered in each round and the number of unclustered sequences in the database. The size of the largest cluster recovered in each round begins to drop with increasing iterations causing the algorithm to spend a lot of time on smaller clusters. We see that, as the number of unclustered sequences decreases, the entropy of the clusters discovered drops indicating that the clusters identified in the later iterations comprise a relatively uniform set of small cluster sizes. Since the size of the clusters still to be identified is expected to be smaller than the largest cluster identified in each round, the odds of sampling sequences from small or singleton clusters increase with increasing iterations. We performed simulations with different probability distributions for the cluster sizes and we see that the entropy of the cluster sizes identified in each round correlates well with the number of unclustered sequences and remains independent of the nature of the distribution. We include similar analysis of cluster sizes sampled from uniform and normal distributions in Supplementary Figure S1 and Section 3.1 of Supplementary Data.

The two lemmas described above as well as Figure 1B and the simulation studies confirm the intuition that, in each iteration, the naive sampling algorithm is likely to identify, and remove from subsequent iterations, the largest clusters in the data set (as per Lemma 2, the probability of any iteration missing all of the large clusters is small). As a result, the set of unclustered sequences becomes increasingly depleted of large clusters, making it less likely that any large clusters will be identified in each iteration for a given sampling rate *α*. To counteract this effect, we describe an adaptive process that increases the value of *α* as the ‘productivity’ of each iteration decreases. The naive sampling approach serves as the lower bound for the performance of SCRAPT as it assumes a constant sampling rate and does not make any assumptions on the choices of centroids.

A more useful measure is an estimate of the number of clusters of interest that is pending to be clustered at the end of each iteration. We show that the problem of determining this probability is computationally hard even if the sizes of all clusters are known in advance. We describe a detailed analysis in Section 1.3 in Supplementary Data (Theorem 1). In this manuscript, we describe a statistical procedure to estimate an upper bound on the size of the unobserved clusters in each iteration.

In the next section, we provide two strategies that improve the naive sampling baseline in terms of efficiency and the quality of the clusters produced.

### Adaptive sampling

To formalize the adaptive sampling, we assume a sample of *n* sequences drawn from unclustered sequences available at the beginning of the iteration. Since the number of singletons in the data set *c* is constant (a true singleton will not be clustered in any iteration), we can write |unclustered| = |non-singletons| + *c* and observe that |non-singletons| decreases from iteration to iteration as clusters are identified. As a result, the ‘density’ of singletons in the data set, ${c}/({|{\rm non}\hbox{-}{\rm singletons}| + c})$, increases during the execution of the algorithm, making it less likely for any subsample to contain two or more sequences from the same cluster. To address this issue, we propose an adaptive sampling process that increases the sampling rate $\alpha = {n}/({|{\rm non}\hbox{-}{\rm singletons}|+ c})$, whenever the number of clusters identified in an iteration drops from the previous iteration. Specifically, we increase the sampling rate by a fraction of the relative difference in the number of sequences clustered in the current iteration and the prior iteration of the algorithm. The details are provided in Algorithm 1.

### Mode shifting

The choice of cluster representative affects the quality of the resulting clusters (27). While DNACLUST and CD-HIT use the longest sequence in each cluster as the cluster representative, such sequence is not necessarily the best representative of the set of sequences in the cluster or necessarily a good approximation of the biological sequence from which the co-clustered sequences were derived (i.e. the sequence the clustering process is meant to approximate). Instead, it has been previously proposed that a better representative is the sequence that has the highest multiplicity (largest number of identical copies in the data set) (28), as it likely represents the oversampling of the correct biological sequence from which the other sequences in the cluster diverged due to errors. The details of the algorithm are included in Section 2 in Supplementary Data.


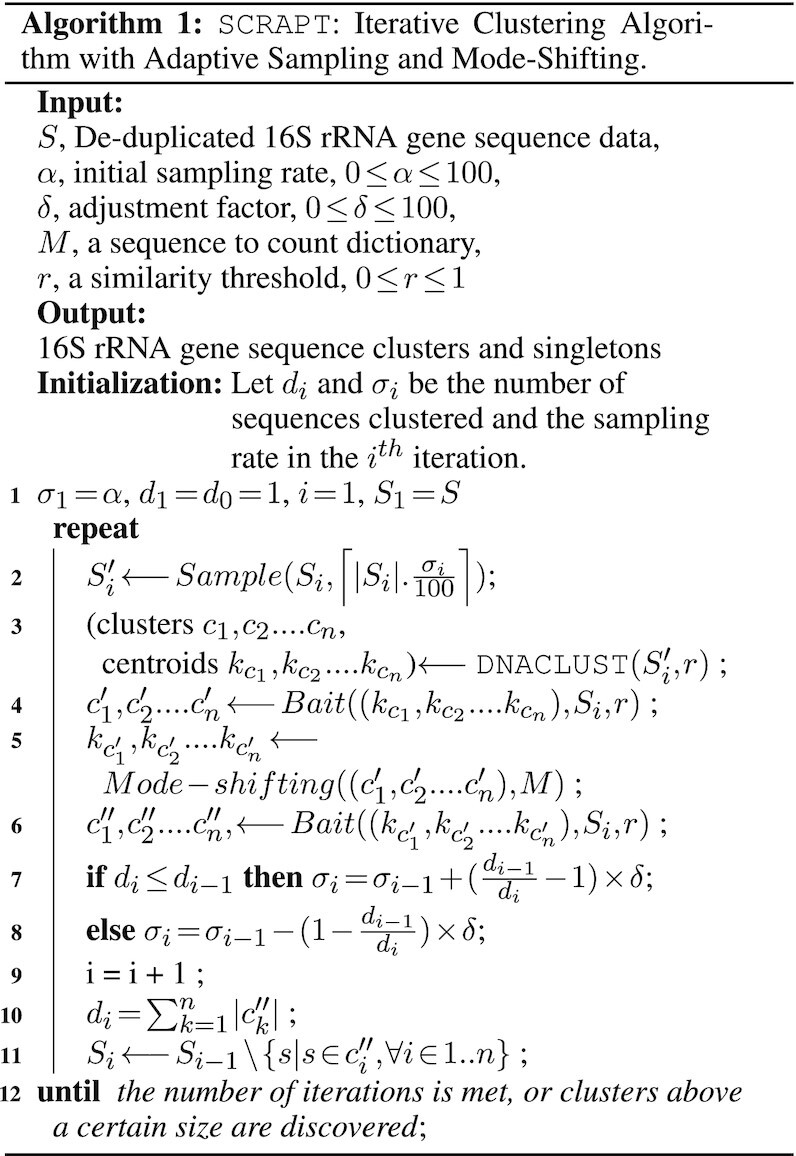


### Estimating confidence intervals for the clusters discovered by SCRAPT

To estimate a bound on the expected number of clusters that are identified in any given round, we need to estimate two probabilities: that of missing any given *m* clusters ($\bar{p}_k^m$) and that of capturing any given *m* clusters ($p_k^m$). We show that the computation of $p_k^m$ is computationally hard (see Theorem 1 in Supplementary Data). We propose a simulation-based procedure to provide an upper bound on the size of the largest cluster discovered in each iteration. Using information about the clusters generated in all prior iterations, at the end of each iteration we estimate the number and size of clusters that may remain within the unclustered sequences. Given a distribution of cluster sizes, we compute the expected number of clusters of a given size *k*, taking into consideration both the clustered and unclustered sequences for that round. For any given size *k*, we simulate the total number of clusters using the calculations shown in Lemma 1. We perform these simulations *r* times and record the clusters discovered in each of the *r* realizations. If the total number of clusters of size *k* discovered in any given iteration matches at least (1 − *ε*) × *r* realizations, where *ε* is a parameter, then we mark all clusters of a given size *k* as having been detected. By default, we set *r* = 1000 and *ε* = 0.05.

SCRAPT uses DNACLUST to cluster a subsample of the data and then uses the cluster representatives as ‘bait’ to retrieve, from the full data set, the other sequences belonging to these clusters. For each cluster obtained after baiting the full data set, we shift the representative sequence to the sequence that has the highest multiplicity in the cluster. The baiting process is repeated after shifting the representative sequence in order to construct the final clusters, potentially recruiting new sequences, as well as returning previously clustered sequences to the unclustered pool. After all clusters shift the representative sequences, SCRAPT repeats the baiting process with the newly selected cluster representatives, to account for any sequences that may need to change their cluster membership. We describe a detailed algorithm to perform mode shifting in Algorithm S1 in Supplementary Data.

## RESULTS

### Experiments

To evaluate the SCRAPT algorithm, we rely on two 16S rRNA gene sequence data sets of varying sizes. The first data set contains 16S rRNA gene sequencing reads of gut microbiome of mice affected with lupus (bioprojects #PRJNA529260 and #PRJNA638971) (29,30). This data set, referred to as the lupus microbiome data set, contains ∼6 million paired-end reads (980 180 reads after deduplication) with an average length of 250 bp after merging. The second data set we have utilized in this study is a soil microbiome data set (bioproject #PRJEB15061) (31) sequenced as a part of the Earth Microbiome Project (32). This data set, referred to as the soil microbiome data set, is deeply sequenced and contains ∼94 million single-end reads (16 608 513 reads after deduplication) with an average length of 150 bp. We compare the performance of the SCRAPT algorithm to several variants—naive sampling without mode shifting, adaptive sampling without mode shifting and naive sampling with mode shifting—as well as against the original DNACLUST. In addition to DNACLUST, we also compare the performance of SCRAPT algorithm to CD-HIT and UCLUST, both of which are popular algorithms to perform sequence clustering. We also compare the cluster representatives produced by SCRAPT to the denoised sample sequences produced by DADA2. Finally, to demonstrate the scalability of SCRAPT, we rely on a large data set of 18S amplicon sequences (bioproject #PRJEB9737), sequenced as a part of the Tara Oceans project (33). This data set comprises 77 329 514 paired-end reads (22 511 580 reads after deduplication), with an average amplicon size of 420 bp. For this experiment, we only compare the clusters produced by SCRAPT to the ASVs produced by DADA2.

We compare these different approaches based on the number of sequences clustered and the distribution of the recovered cluster sizes. Since all these methods, except DADA2, take as input the percentage of similarity that needs to be preserved between the representative sequence to any sequence within the cluster, a clustering algorithm that produces fewer clusters for a given similarity threshold is preferred over others, and is expected to be more accurate. While just the number of clusters might not sufficiently depict the extent of fragmentation, we propose a precise metric that reflects the distribution of cluster sizes.

#### Fragmentation measure

We compute the extent of fragmentation as follows: we order all the clusters in decreasing order of their sizes and, for every size *x*, we measure the total number of sequences contained in clusters of size *x* or greater. This mirrors approaches used to evaluate the fragmentation of genome assemblies (34,35). Throughout the following, we ignore singleton clusters. We refer to this curve as the fragmentation measure in the rest of the document. While plotting the fragmentation measure between two clustering methods, the clustering method whose curve is at the top is said to be less fragmented than the other.

We evaluate all the above-mentioned algorithms for similarity thresholds of 95%, 96%, 97%, 98% and 99%, but for brevity, we discuss the results obtained with a similarity threshold of 98% for the lupus microbiome data set and 97% for the soil microbiome data set. For variants of our iterative algorithms, we studied their respective performances for an initial sampling rate *α* of 0.1%, 0.5%, 1%, 5% and 10% for the lupus microbiome data set and 0.01%, 0.05%, 0.1%, 0.5% and 1% for the soil microbiome data set. We show that SCRAPT is efficient even for small values of *α*.

### Evaluation of SCRAPT against other iterative clustering strategies

**Figure 2.**
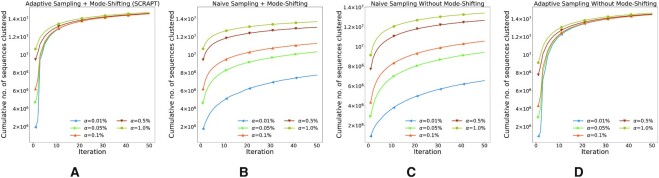
The cumulative number of sequences clustered by (**A**) SCRAPT,**(B)** naive sampling (fixed *α*) with mode shifting, (**C**) naive sampling (fixed *α*) without mode shifting and (**D**) adaptive sampling without mode shifting, for different values of *α* on the soil microbiome data set.

**Figure 3.**
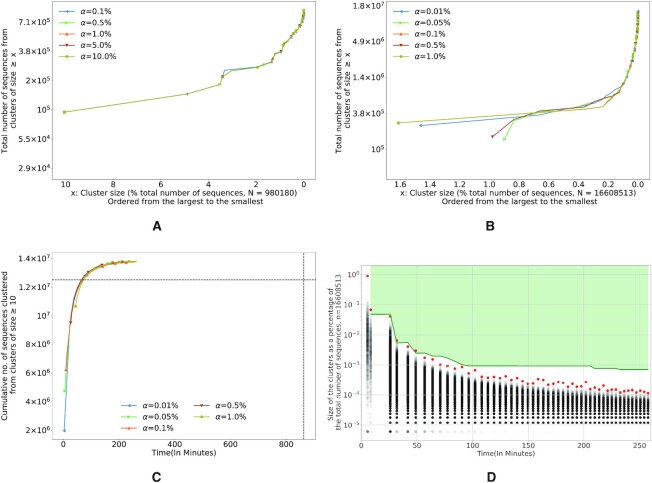
(**A**, **B**) Measure of fragmentation of SCRAPT for different initial sampling rates, *α* (higher the curve, lesser is the extent of fragmentation) for the lupus microbiome data set and the soil microbiome data set. (**C**) Time taken per iteration to identify clusters of size at least 10 on the soil microbiome data set. The dashed lines represent the number of sequences belonging to clusters of size at least 10 and the total time taken by DNACLUST when run stand-alone. (**D**) Distribution of cluster sizes on the soil microbiome data set for the clusters discovered by each round of SCRAPT with time along the *x*-axis. The red dots indicate the size of the largest cluster identified in each iteration. The shaded region represents the confidence interval identified by the bootstrapping procedure of SCRAPT, specifically an upper bound on the size of clusters that may be detected in that iteration.

In Figure 2, we compare SCRAPT to three variants of the iterative clustering approach (adaptive sampling without mode shifting, naive sampling with mode shifting and naive sampling without mode shifting) on the soil microbiome data set. We show a similar figure for the lupus microbiome data set in Supplementary Figure S2. As seen from Figure 2C, the naive iterative clustering with a fixed sampling rate and without mode shifting requires a larger sampling rate in order to cluster most of the data set—clustering quickly reaches a fairly low plateau for low values of *α*. A low value of *α* is desirable as the run time of the clustering algorithm critically depends on the size of the subsample selected in each iteration. Mode shifting (Figure 2B) and the adaptive sampling rate (Figure 2D) each partly overcome this effect; however, their combination (SCRAPT, Figure 2A) achieves the best performance even at lower sampling rates and the number of sequences clustered remains independent of *α*. In Figure 3A and B, we compare the distribution of the cluster sizes recovered by SCRAPT at different values of *α* for the lupus and soil microbiome data sets, respectively. As can be seen from the figure, the cluster distributions are indistinguishable, despite the different convergence rates for different starting sampling rates *α*, particularly for the larger clusters (left side of the figure). We also provide a comparison of the mode-shifting process used in SCRAPT with the longest-sequence-first approach used by CD-HIT and DNACLUST in Supplementary Figure S4.

To better exemplify the clustering process, we show the relationship between run time and the cumulative sequences clustered in the soil microbiome data set in Figure 3C. We show the number of sequences clustered by DNALCUST in stand-alone mode, in dashed lines, to provide a frame of reference. All iterative approaches, irrespective of the value of *α*, complete the clustering faster than the original DNACLUST—a side effect of the mode-shifting approach, demonstrating that the choice of the longest sequence as cluster representative as followed by DNACLUST is suboptimal. We also restricted the analysis to clusters of size at least 10 and we see that the iterative approach for different values of starting *α* quickly converges, indicating SCRAPT is able to identify all clusters of size at least 10 early on, independent of the choice of *α*. In Figure 3D, we plot the distribution of cluster sizes identified in each iteration on the soil microbiome data set, with the largest cluster highlighted in red. As seen in this figure, the largest clusters in the data set are rapidly detected in the first few iterations, after which the clustering process reaches a plateau during which each iteration identifies a subset of the (large number of) small clusters in the data set. Depending on application, the clustering process may, thus, be terminated as soon as the relevant clusters have been identified. The *x*-axis in Figure 3D represents the time spent by the algorithm to reach a particular iteration, indicating that within 150 min, the clustering process has identified all clusters of size at least 50, or 0.0002% of the total number of sequences. For the want of space, we have included results from the lupus microbiome data set in Supplementary Figure S3. Despite differences in data sets, the comments we make on the larger data set hold on the smaller data set as well.

### Evaluation of SCRAPT against other clustering strategies

#### SCRAPT produces less fragmented clusters

**Figure 4.**
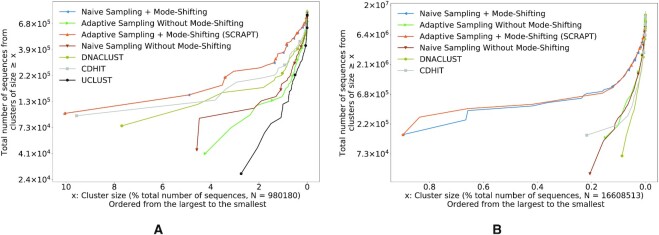
Comparing the fragmentation measure of different variations of the iterative clustering: SCRAPT, adaptive sampling without mode shifting, naive sampling with and without mode shifting and other stand-alone clustering tools: DNACLUST, CD-HIT and UCLUST (higher the curve, lower is the fragmentation). The curve for naive sampling with mode shifting (shown in blue) is hidden behind the curve for adaptive sampling with mode shifting (SCRAPT shown in orange). (**A**) Fragmentation curves for the lupus microbiome data set for a similarity of 0.98. (**B**) Fragmentation curves for the soil microbiome data set for a similarity of 0.97.

In Figure 4A and B, we study the quality of clusters produced by different methods on the lupus and soil microbiome data sets, respectively. We plot the extent of fragmentation for different variations of the iterative clustering algorithm and the stand-alone versions of DNACLUST, CD-HIT and UCLUST. From the figures we see that the extent of fragmentation is very low for SCRAPT and the iterative algorithm that uses a fixed sampling rate (the naive method). This is an artifact of the mode-shifting procedure and confirms our intuition that the mode is closer to the ‘true centroid’ of the cluster. However, as described previously, the naive sampling tends to converge sooner and might fail to identify all large clusters for stringent similarity thresholds as it tends to spend a lot of time detecting singletons in the later iterations. Similarly, we see that the variations of the iterative algorithm that do not perform mode shifting tend to produce low-quality clusters. With respect to other stand-alone clustering tools, we see that none of the existing methods outperform SCRAPT and the clusters produced by DNACLUST, CD-HIT and UCLUST are more fragmented. We do not show the results of running UCLUST on the soil data set because the open-source version of UCLUST failed to produce outputs on these data. We also study the performance of SCRAPT against MeShClust 3.0 (36) on the lupus microbiome data set and have included results in Supplementary Figure S5 and Section 3.5 of Supplementary Data. MeShClust considers some sequences as ‘extended members’ of clusters. These are sequences that do not respect the input similarity threshold with respect to the centroid but have a very low classification error. The fragmentation measure of the clusters produced by MeShClust is comparable to SCRAPT when considering the extended members; however, SCRAPT runs orders of magnitude faster than MeShClust.

#### SCRAPT is faster than other popular methods

**Figure 5.**
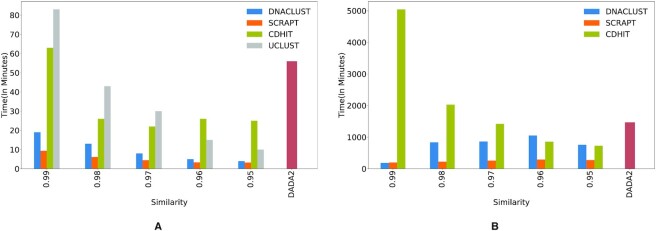
Comparing the run times of SCRAPT and other OTU clustering tools—CD-HIT, UCLUST and DNACLUST for different similarity thresholds for (**A**) the lupus microbiome data set and (**B**) the soil microbiome data set. (The results from UCLUST for the soil microbiome data set are not shown because the open-source version of UCLUST failed to produce outputs on this data set.)

Finally, we describe the run time benchmarks for SCRAPT, DNACLUST, CD-HIT, UCLUST and DADA2 for the lupus and soil microbiome data sets in Figure 5A and B, respectively. We see that the run time of SCRAPT is independent of the similarity threshold while all the other tools took times proportional to the stringency of the similarity thresholds. All the above-mentioned tools were run with 8 threads on a Xeon E5-2680 machine with a maximum memory allocation of 36 GB. We note that SCRAPT required much lower memory (1.2 GB on an average) and that can be attributed to DNACLUST’s memory frugality.

#### SCRAPT and DADA2 produce a similar set of clusters

**Figure 6.**
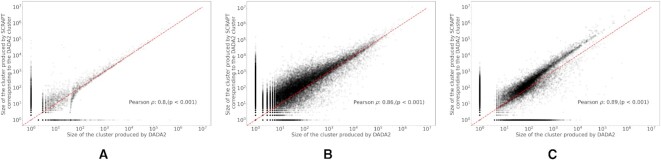
Comparison of clusters produced by DADA2 and SCRAPT on (**A**) the lupus microbiome data set, (**B**) the soil microbiome data set and (**C**) the Tara Oceans data set.

We assess how similar the output of SCRAPT is to DADA2’s output, which is currently the widely accepted standard for 16S rRNA gene sequence analysis. DADA2 takes the entire data set (without deduplication) as an input and outputs the ‘top’ denoised sample sequences along with the number of sequences within the ASV, which is used for downstream analysis tasks. To compare the quality of the clusters produced by SCRAPT, we took the centroids obtained from the clusters of SCRAPT and aligned them against the denoised sequences of DADA2. For each denoised sequence identified by DADA2, we found the largest SCRAPT centroid that had at least 99% similarity with the former. We then computed the number of sequences within the corresponding SCRAPT cluster for each ASV associated with DADA2. In Figure 6, we show the relationship between the cluster sizes of DADA2 and the number of sequences in the largest cluster of SCRAPT corresponding to each DADA2 cluster for the data sets considered in this study. It is evident from the plot that the sizes of the clusters discovered by both the methods are highly correlated (Pearson *ρ* = 0.80, *P* < 0.001, Pearson *ρ* = 0.86, *P* < 0.001 and Pearson *ρ* = 0.89, *P* < 0.001 on the lupus microbiome data set, soil microbiome data set and Tara Oceans data set, respectively). Further, we note that a few clusters are identified only by SCRAPT and DADA2, respectively. On the lupus microbiome data set, 5.5% and 2.24% sequences are clustered only by SCRAPT and DADA2, respectively. Similarly, on the soil microbiome data set, 8.56% and 0.05% of the sequences are clustered only by SCRAPT and DADA2, respectively. Finally, on the Tara Oceans data set, only 0.64% and 1.55% of the sequences are clustered exclusively by SCRAPT and DADA2, respectively. This indicates that the two methods agree on the choice of centroids to a large extent. We also see that SCRAPT is able to produce larger clusters with more sequences in them than DADA2. We have also computed the fragmentation measure between DADA2 and SCRAPT and have included the fragmentation curves (Supplementary Figure S6) in Section 3.6 of Supplementary Data. Although both SCRAPT and DADA2 produce similar clusters, in our experiment for the same data set we see that the SCRAPT runs faster than DADA2. The run times for the lupus microbiome and soil data sets are shown in Figure 5. On the Tara Oceans data set, SCRAPT took 171 min, which is 27× faster than DADA2, which took 4677 min. We also note that we ran DADA2 in its default sample-by-sample mode, while we ran SCRAPT by pooling all sequences from all samples, since DADA2 would not complete in a reasonable amount of time when applied to pooled data for the soil and Tara Oceans data sets.

## DISCUSSION

In this manuscript, we have described an iterative approach for sequence clustering, implemented in the software SCRAPT, which we evaluate in the context of 16S rRNA gene sequence analyses—a widely used approach for assessing the structure of microbial communities. Our results show that iterative clustering achieves a substantial improvement in performance over one-shot clustering approaches such as DNACLUST even without the enhancements described in our paper. We also provide a theoretical analysis of the iterative clustering process that can be used to provide guaranteed bounds on the size of clusters that can be identified in each clustering round.

Results also show diminishing returns as clustering proceeds, with later iterations of the clustering process identifying only relatively small clusters. This provides the opportunity for users to trade off analysis time for the completeness of the clustering, because small clusters are often due to errors or contain insufficient information to allow meaningful subsequent statistical analyses.

In general, the quality of clusters produced by SCRAPT is not sensitive to the choice of initial sampling rate *α*, as shown in Figure 3A: the curves for different initial sampling rates agree with respect to the fragmentation measure. However, we note that in Figure 3B, the curves start at different positions along the *x*-axis for the soil data set, suggesting that the initial sampling rate *α* could have an effect on the size of the largest clusters discovered. Such an observation can be explained by the relative noise content (i.e. the sequences lie in between real clusters) in the data set that causes the algorithm to split a large cluster. However, as indicated in Figure 4, SCRAPT still outperforms the existing methods by a large margin, and the difference caused by different initial sampling rates *α* in SCRAPT is negligible in this context.

We also observe from our experiments that reassigning cluster representatives to sequences with highest multiplicity yields a significant improvement in the size of clusters that are recovered, while preserving the similarity within the clusters. It is important to highlight that while other studies have shown the advantage of using sequence multiplicity to guide the clustering, many clustering approaches continue to rely on a ‘longest-sequence-first’ paradigm. In our case, the mode-shifting procedure operates in just one round (sequences are ‘baited’ once after the reassignment of the cluster representative); however, we have noticed that some clusters could be further improved by multiple rounds of mode shifting. An exploration of the context within which it would be beneficial to repeatedly ‘recenter’ clusters is left for future research.

The fragmentation measure for the lupus microbiome data set (Figure 4A) indicates that the performance of CD-HIT is comparable to SCRAPT for this data set, while in the soil microbiome data set (Figure 4B) CD-HIT leads to more fragmented results. We believe this behavior is due to the greedy fashion in which CD-HIT ‘seeds’ the clusters. The representative sequence for each cluster is simply the longest sequence that could not be assigned to any other cluster. The length of sequences can be influenced by experimental factors (e.g. sequencing errors or incorrect trimming of adapters can result in lengthened sequences), thus leading to inconsistent performance from sample to sample. The mode-shifting approach used by SCRAPT addresses this confounding factor, leading to a more consistent behavior across samples.

The state-of-the-art tools for 16S rRNA gene sequence analysis, including CD-HIT, UCLUST, DADA2 and DNACLUST, rely on sequence alignment. Since our focus is on 16S rRNA gene data sets, we illustrate and compare the performance of SCRAPT with tools that use alignments. An important factor that enables the iterative approach we have described here is the ‘baiting’ procedure used by DNACLUST wherein the clustering algorithm identifies a potential cluster representative from subsample and then searches the rest of the data set for other sequences that lie within the similarity radius of the representative. That said, the iterative clustering scheme presented in this paper can be easily extended to any of the existing clustering tools, including BLAST and alignment-free tools as long as baiting and centroid-shifting functions can be defined.

The authors of DADA2 claim that DADA2 is able to better capture true biological signals than OTU-based approaches. Our comparison between SCRAPT and DADA2 reveals high concordance between the output of the two tools (Figure 6), demonstrating that a carefully designed OTU clustering approach can match the accuracy of the statistical approach implemented in DADA2 while significantly improving the analysis run time. The correlation between cluster sizes shows that there is an agreement on cluster centroids produced by DADA2 and SCRAPT. This further shows that DADA2 and SCRAPT produce a highly similar set of clusters and the improved efficiency of SCRAPT does not trade off the quality of clusters produced by SCRAPT. It further confirms that the mode of clusters, chosen by SCRAPT as the reassigned centroid, is a biologically relevant estimator of the cluster centroid while incurring a low computational cost.

The efficiency of SCRAPT comes from its ability to prioritize the discovery of larger clusters in the first few rounds, making the subsequent iterations cheap. Further improvements in speed can be obtained through parallelization. Specifically, within each iteration, after ‘seeding’ the clusters, the bait, mode-shift, rebait process can be run independently and in parallel for each cluster. In order to remain consistent with how other clustering tools operate, we have not leveraged this opportunity for the results presented in this manuscript.

It is well appreciated that using a fixed sequence similarity threshold in OTU clustering can cause inflation in community richness (37). Since the current implementation of SCRAPT also relies on a fixed sequence similarity threshold, our tool also suffers from this limitation. However, we believe that the mode-shifting process at least partly alleviates the impact of the potentially more fragmented clustering results. Mode shifting focuses on the ‘denser’ regions of the sequence space, meaning that fewer sequences fall out of range, and also that more of the sequences are assigned to the proper clusters. As a result, the potentially spurious clusters caused by the strict identity threshold encompass fewer sequences and are overall smaller in size. Thus, the fragmentation is unlikely to impact most downstream analyses of the data, beyond the obvious impact on richness measures. The strong concordance between cluster abundances observed when comparing SCRAPT with DADA2 supports this conjecture. We are, however, interested in further refining the clusters produced by SCRAPT. Co-abundance information, for example, can be used to expand the clustering threshold in a data-driven manner, as done in LULU (38). We also believe, however, that the clusters produced by SCRAPT and other fixed-threshold tools can also be underfragmented. Approaches such as the divisive clustering used in DADA2 can be used to break apart clusters in a data-dependent way. Such future research must, however, be performed in the context of relevant biological applications to ensure that the clustering results enhance rather than confound analyses.

## CONCLUSION

In this paper, we study the benefits of adopting an iterative clustering framework in the context of clustering large 16S rRNA gene data sets. However, the background and the theory we have described in the previous sections do not make any assumptions on the nature of the data or the process that generated the data. With an appropriate distance function, a centroid-shifting function and a baiting function, the iterative approach can be extended to other kinds of clustering problems in general. Conversely, assumptions about the nature of the data or the process that generated them can allow us to speed up the clustering process by biasing the sampling toward sequences belonging to large clusters. This serves as the focus of future research.

As discussed, there are opportunities for extending the iterative clustering framework we have described to other clustering algorithms and sequence search approaches. We intend to evaluate how the choice of such algorithms impacts the effectiveness and efficiency of the SCRAPT approach. We also note that this manuscript has primarily provided a comparison of SCRAPT to the non-iterative application of DNACLUST, CD-HIT and UCLUST. We plan to conduct a more thorough comparison of SCRAPT to other widely used clustering tools, including alignment-free methods. Currently, SCRAPT relies on uniform sampling of the data. We plan to explore in the future biased sampling procedures that increase the likelihood that large clusters found in the subsamples are selected in each iteration. Such biased sampling approaches may be particularly effective in the later stages of the clustering process where, even after adaptively changing the sampling rate, the clustering process stalls. Studies (39) in the past have shown that using fixed thresholds often misses out on useful information. Additionally, the advent of long-read sequencing technologies has made it possible to sequence the entire gene as opposed to just a region of the 16S rRNA gene. Research (40) also shows that analysis involving the entire gene improves the taxonomic characterization of the data. We hope to incorporate a support for full-length gene sequences in the future versions of SCRAPT.

## DATA AVAILABILITY

The algorithm is implemented in the open-source package SCRAPT. The source code used to generate the results presented in this paper is available at https://github.com/hsmurali/SCRAPT and https://doi.org/10.5281/zenodo.7659103.

## Supplementary Material

gkad158_Supplemental_File
