# Supplementary material for: SCRAPT: an iterative algorithm for clustering large 16S rRNA gene data sets
Source: Nucleic Acids Res. 2023 Mar 13;51(8):e46. doi: 10.1093/nar/gkad158 (PMC10164572; doi:10.1093/nar/gkad158)
Supplement: gkad158_Supplemental_File [file gkad158_supplemental_file.pdf]

SCRAPT: An Iterative Algorithm for Clustering Large 16S rRNA Gene Data Sets

Tu Luan [1,2#], Harihara Subrahmaniam Muralidharan [1,2#], Marwan Alshehri [1], Ipsa Mittra [1], and Mihai Pop [1,2*]

1 Proofs of the lemmas

1.1 Lemma-1

Lemma 1: Given N sequences and a sample obtained by uniformly sampling n sequences, the bound on the probability of missing a cluster c_k of size ρ_k is given by,

$$\bar{p}_k = \frac{\binom{N-\rho_k}{n-1}.\rho_k}{\binom{N}{n}} + \frac{\binom{N-\rho_k}{n}}{\binom{N}{n}} \leq e^{-\frac{\rho_k n}{N}}(1 + \frac{\rho_k n}{N} e^{\frac{\rho_k}{N}}).$$

Proof. The proof the equality in the lemma follows from the fact that, the cluster of size ρ_k is missed only if either of the following two cases happen: 1. there is exactly one sequence from cluster k present in the sample 2. no sequences from cluster k is present in the sample. Thus, adding the two we get the probability of missing a cluster as,

$$\bar{p}_k = \frac{\binom{N-\rho_k}{n-1}.\rho_k}{\binom{N}{n}} + \frac{\binom{N-\rho_k}{n}}{\binom{N}{n}} \tag{1}$$

The second part of the inequality comes from approximating Equation 1.

It is a well known fact that, $\binom{n}{k} \leq \frac{n^k}{k!}$

$$\implies \frac{\binom{N-\rho_k}{n-1}}{\binom{N}{n}} \approx \frac{\frac{(N-\rho_k)^{n-1}}{(n-1)!}.\rho_k}{\frac{N^n}{n!}} = \frac{(N-\rho_k)^{n-1}.\rho_k.n}{N^n}$$

$$\frac{\binom{N-\rho_k}{n}}{\binom{N}{n}} \approx \frac{(N-\rho_k)^n}{N^n}$$

$$\bar{p}_k = \frac{(N-\rho_k)^{n-1}.\rho_k.n}{N^n} + \frac{(N-\rho_k)^n}{N^n}$$

$$= (1-\frac{\rho_k}{N})^{n-1}(1-\frac{\rho_k}{N}+\frac{n\rho_k}{N})$$

$$= (1-\frac{\rho_k}{N})^{n} + \frac{n\rho_k}{N}(1-\frac{\rho_k}{N})^{n-1}$$

Applying the Approximation, $(1-x)^r \leq e^{-xr}, \forall 0 \leq x \leq 1$

$$\bar{p}_k \leq e^{-\frac{n\rho_k}{N}} + \frac{n\rho_k}{N}e^{-\frac{\rho_k(n-1)}{N}} = e^{-\frac{n\rho_k}{N}}(1+\frac{n\rho_k}{N}e^{\frac{\rho_k}{N}})$$

□

1.2 Lemma-2

Lemma 2: Given N sequences in the database with n sequences in the sample the probability $\bar{p}_k^m$ of missing m clusters of size $k = [\rho_1, \rho_2, .., \rho_m]$ such that $\rho_1 \leq \rho_2 \leq ... \leq \rho_m$ is given by,

$$\bar{p}_k^m = \sum_{i=0}^{m} B_{m,i} * \frac{\binom{N-\sum_{j=1}^{m}\rho_k^j}{n-i}}{\binom{N}{n}} \leq e^{-\frac{m\rho_k^1 n}{N}}(1+\frac{\rho_k^1 n}{N}e^{\frac{m\rho_k^1}{N}})^m; \text{where, } B_{i,j} = \begin{cases} 0 & \text{if } i=0 \\ B_{i-1,j-1}k_i + B_{i-1,j} & \text{otherwise} \end{cases}$$

Proof. We begin by noting that sampling m clusters of sizes ρ_1 is not independent of one another as they are dependent on the number of sequences sampled notated by n. Hence the probabilities of missing one cluster of size ρ_k as given by Lemma 1 cannot be multiplied. Expanding the summation on we get the following,

$$\bar{p}_k^m = \frac{\binom{N-K_m}{n}}{\binom{N}{n}} + \frac{\binom{N-K_m}{n-1}}{\binom{N}{n}}(\rho_1 + \rho_2 + .. + \rho_m) + \frac{\binom{N-K_m}{n-2}}{\binom{N}{n}}(\rho_1\rho_2 + \rho_1\rho_3 + .. + \rho_{m-1}\rho_m) + .. + \frac{\binom{N-K_m}{n-m}}{\binom{N}{n}}(\rho_1.\rho_2...\rho_m);$$
$$\text{where, } K_m = \sum_{i=1}^{m} \rho_i$$

Remember that a cluster is missed from being identified in a certain round if it has either 0 or 1 representatives in the sample. The first term refers to the case when no sequences are sampled from each of the m clusters and the second term refers to the case where exactly one sequence is sampled from 1 of the m clusters and the other respective $m-1$ clusters have 0 representatives and so on. We prove that this statement holds good by an induction on the number of clusters m.

Base Case: Lets us show that the statement holds good for the smallest m, $m = 1$.

$$\bar{p}_1 = \frac{\binom{N-\rho_1}{n}}{\binom{N}{n}} + \frac{\binom{N-\rho_1}{n-1}}{\binom{N}{n}}\rho_1 \leq e^{-\frac{\rho_1 n}{N}}(1 + \frac{\rho_1 n}{N}e^{\frac{\rho_1}{N}})$$

We know that this true which is a consequence of lemma 1, $\bar{p}_1$ is the probability of missing one cluster of size ρ_1.

Inductive Step: Since it holds good for $m = 1$ let us assume that this statement is true for m clusters. Lets us calculate the odds of missing $m+1$ clusters. The odds of missing $m+1$ clusters is simply the odds of missing m clusters and missing one other cluster of size ρ_{m+1}. The cluster $m+1$ is also missed if either 0 or 1 sequence from the cluster is a part of the sample. Therefore we get,

$$\bar{p}_k^{m+1} = \frac{\binom{N-K_m}{n}}{\binom{N}{n}}\left(\frac{\binom{N-K_{m+1}}{n}}{\binom{N-K_m}{n}} + \frac{\binom{N-K_{m+1}}{n-1}}{\binom{N-K_m}{n}}\rho_{m+1}\right) + \frac{\binom{N-K_m}{n-1}}{\binom{N}{n}}(\rho_1 + \rho_2 + .. + \rho_m)\left(\frac{\binom{N-K_{m+1}}{n}}{\binom{N-K_m}{n-1}} + \frac{\binom{N-K_{m+1}}{n-1}}{\binom{N-K_m}{n-1}}\rho_{m+1}\right)$$
$$+ \frac{\binom{N-K_m}{n-2}}{\binom{N}{n}}(\rho_1\rho_2 + \rho_1\rho_3 + .. + \rho_{m-1}\rho_m)\left(\frac{\binom{N-K_{m+1}}{n}}{\binom{N-K_m}{n-2}} + \frac{\binom{N-K_{m+1}}{n-2}}{\binom{N-K_m}{n-1}}\rho_k^{m+1}\right) + ... + \frac{\binom{N-K_m}{n-m}}{\binom{N}{n}}(\rho_1.\rho_2...\rho_m)$$
$$\left(\frac{\binom{N-K_{m+1}}{n}}{\binom{N-K_m}{n-m}} + \frac{\binom{N-K_{m+1}}{n-m}}{\binom{N-K_m}{n-m}}\rho_k^{m+1}\right); \text{ where, } K_m = \sum_{i=1}^{m}\rho_i \text{ and } K_{m+1} = \sum_{i=1}^{m+1}\rho_i$$
$$= \frac{\binom{N-K_{m+1}}{n}}{\binom{N}{n}} + \frac{\binom{N-K_{m+1}}{n-1}}{\binom{N}{n}}(\rho_1 + \rho_2 + .. + \rho_{m+1}) + \frac{\binom{N-K_{m+1}}{n-2}}{\binom{N}{n}}(\rho_1\rho_2 + \rho_1\rho_3 + .. + \rho_m\rho_{m+1}) + .. +$$
$$\frac{\binom{N-K_m}{n-(m+1)}}{\binom{N}{n}}(\rho_1.\rho_2...\rho_{m+1})$$

Given that $\rho_1 \leq \rho_2 \leq ... \leq \rho_{m+1}$ we can say that the probability of missing $m+1$ clusters of size atleast ρ_1 can be upper bounded by the probability of missing $m+1$ clusters of size ρ_1. Hence we get that,

$$\bar{p}_k^{m+1} \leq \frac{\binom{N-(m+1)\rho_1}{n}}{\binom{N}{n}} + \frac{\binom{N-(m+1)\rho_1}{n-1}}{\binom{N}{n}}(m+1)\rho_1 + \frac{\binom{N-(m+1)\rho_1}{n-2}}{\binom{N}{n}}\binom{m+1}{2}(\rho_1)^2 + .. + \frac{\binom{N-(m+1)\rho_1}{n-(m+1)}}{\binom{N}{n}}(\rho_1)^{m+1}$$

Applying, $\binom{n}{r} \leq \frac{n^r}{r!}$ and $(1-x)^r \leq e^{-xr}$,

$$\leq e^{-\frac{(m+1)\rho_1 n}{N}} + \binom{m+1}{1}\frac{\rho_1 n}{N}e^{-\frac{(m+1)\rho_1(n-1)}{N}} + \binom{m+1}{2}(\frac{\rho_1 n}{N})^2 e^{-\frac{(m+1)\rho_1(n-2)}{N}} + ... + (\frac{\rho_1 n}{N})^{m+1}e^{-\frac{(m+1)\rho_1(n-m-1)}{N}}$$
$$= \left(e^{-\frac{n\rho_1}{N}}(1 + \frac{\rho_1 n}{N}e^{\frac{(m+1)\rho_1}{N}})\right)^{m+1}$$

Conclusion: Since both the base case and the inductive step have been proved as true, by mathematical induction the statement $\bar{p}_m$ holds for every whole number m. □

1.3 Theorem-1

Given N sequences in the database, computing the probability p_k^t of capturing t clusters of size at least k, is NP-complete.

Proof. Define the event and the probability of capturing cluster i as c_i and $P(c_i)$ respectively. Define the event and the probability of missing cluster i as $\bar{c_i}$ and $P(\bar{c_i})$ respectively. The proof of the hardness of p_k^t is given by expanding p_k^t into probability form and apply the Inclusion–Exclusion Principle:

$$
\begin{aligned}
p_k^t &= P(c_1 \cap c_2 \cap ...c_t) \\
&= 1 - P(\bar{c_1} \cup \bar{c_2} \cup ...\bar{c_t}) \\
&= 1 - \Big(\sum_{1 \le i \le t} P(\bar{c_i}) - \sum_{1 \le i \le j \le t} P(\bar{c_i} \cap \bar{c_j}) + \sum_{1 \le i \le j \le k \le t} P(\bar{c_i} \cap \bar{c_j} \cap \bar{c_k}) ... + (-1)^{t-1} * P(\bar{c_1} \cap \bar{c_2} ... \bar{c_t}) \Big)
\end{aligned}
$$

In order to compute the second term of the expanded formula, we need to compute the probabilities of a list of events and a list of event intersections, which is an enumeration of $\mathcal{P}(\{\bar{c_1}, \bar{c_2}, ...\bar{c_t}\})$, the power set of event set $\{\bar{c_1}, \bar{c_2}, ...\bar{c_t}\}$. Note that by applying Lemma-2, each individual probability term $P(c_i \cap c_j \cap ...)$ can be computed exactly, however, to compute p_k^t, the total number of probabilities terms we compute will be $|\mathcal{P}(\{\bar{c_1}, \bar{c_2}, ...\bar{c_t}\})| = 2^t$, and therefore the computation of p_k^t is NP-complete. □

2 Mode-shifting Algorithm

Algorithm 1: Mode-shifting

Input: clusters $\{c_1, c_2...c_n\}$, dictionary of counts for sequences–counts, M
Output: a set of representatives $\{k_1, k_2....k_n\}$, corresponding to $\{c_1, c_2...c_n\}$
Data: 16S rRNA sequences gene data set

```
1 Function Mode-shifting(({c1, c2....cn}),M)
2     for ci ∈ {c1, c2...cn} do
3         for a sequence sj, s.t. sj ∈ ci, if M[sj] ≥ M[sk], s.t. ∀k ∈ range(m) \ j, then assign ki = sj to be the
          new representatives for ci
4     return {k1, k2, ...., km}
```

3 Additional Results

3.1 Simulation Analysis

We studied the clustering algorithm using simulations since the distributions of cluster sizes were not known before hand. To that end, as mentioned in the main document, we assumed different distributions on the cluster sizes and studied the relationship between the entropy of the cluster sizes and the number of sequences pending to be clustered in the database. We observed that both the entropy dropped with decreasing number of sequences and their relationship remained independent of the nature of the distribution of clusters. In the main document, we showed plots where we assumed a geometric distribution on the cluster sizes. We show results pertaining to truncated normal distribution(figure S1a and S1b) and uniform distribution (figure S1c and S1d), as an extreme case.

3.2 Evaluating the performance of `SCRAPT` on lupus microbiome data set

As described in the main document, we have included a few results on the lupus microbiome data set for the want of space. We chose to not include them in the main document as they conveyed little new information.

3.3 Performance of `SCRAPT` for different similarity thresholds

We studied the performance of `SCRAPT` with different similarity thresholds. `SCRAPT` consistently outperforms all the other tools considered in this study, independent of similarity thresholds. However, for the want of space we

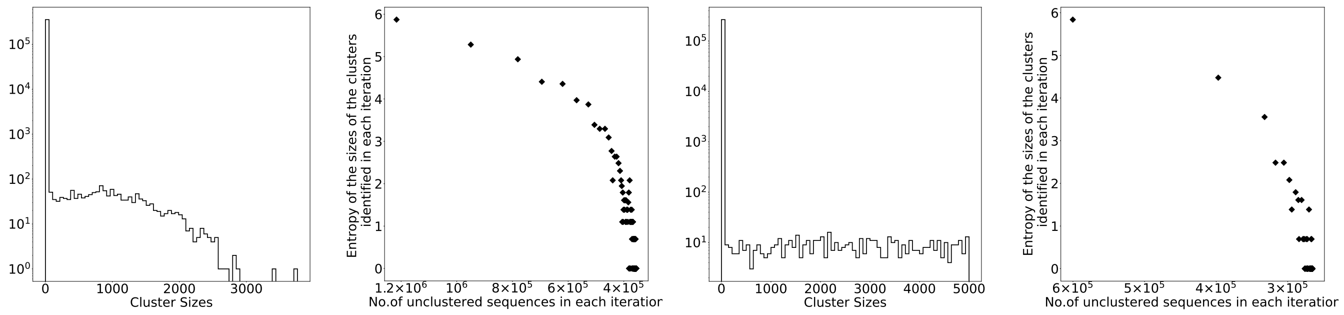

(a) (b) (c) (d)

Figure S1: (a) Distribution of cluster sizes assuming a truncated normal distribution. (b) Entropy of the clusters discovered in each iteration versus the number of unclustered sequences at the end of each iteration for a truncated normal distribution. (c) Distribution of cluster sizes assuming a uniform distribution. (d) Entropy of the clusters discovered in each iteration versus the number of unclustered sequences at the end of each iteration for a uniform distribution.

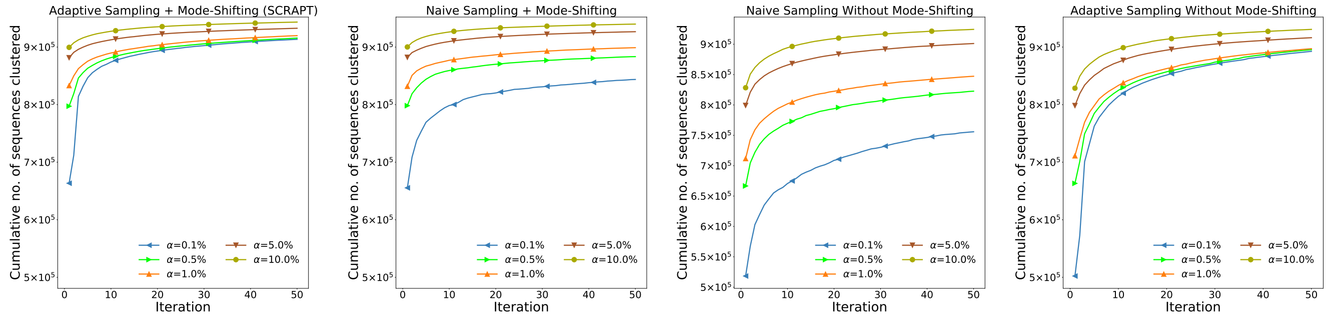

(a) (b) (c) (d)

Figure S2: The cumulative number of sequences clustered by (a) SCRAPT (b) Naive sampling (fixed α) with mode-shifting) (c)Naive sampling (fixed α) without mode-shifting (d) Adaptive sampling without mode-shifting, for different values of α on the lupus microbiome data set for a similarity of 0.98.

describe our results and interpretations only for a similarity threshold of 98%(0.98). For the all the similarity thresholds the results and the conclusions we made hold true. The results for all other similarities for each of the data sets we have taken up in this study can be found here: https://drive.google.com/drive/folders/1n976A2n18WUg1jJrSXNZEFUz559jwLB3?usp=sharing

3.4 Comparing mode against the longest sequence for centroid

We studied the sequence with the largest length against mode of the cluster. For a similarity threshold of 0.98 and an initial sampling rate of 0.1, we ran SCRAPT with the sequence with the highest multiplicity(mode) within a cluster as a choice of centroid and the sequence with the greatest length as a choice of centroid and compared their clustering qualities. We show the cumulative number of sequences clustered and the measure of fragmentation in figure figure S4. We see from the figures that SCRAPT with mode clearly as the centroid outperforms SCRAPT with the longest sequence as the centroid. SCRAPT with mode as centroid produces larger and less fragmented clusters for the same choice of initial clustering parameters reinforcing our intuition that the mode is a better choice of centroid than length.

3.5 Comparing SCRAPT Against MeShClust 3.0

We compare SCRAPT against MeShClust 3.0 which uses a sampling and mean-shifting based approach to perform sequencing clustering. We computed the fragmentation measure between SCRAPT and MeShClust 3.0 and further benchmarked the run-times. MeShClust 3.0 places sequences under three categories, M, sequences belonging to a

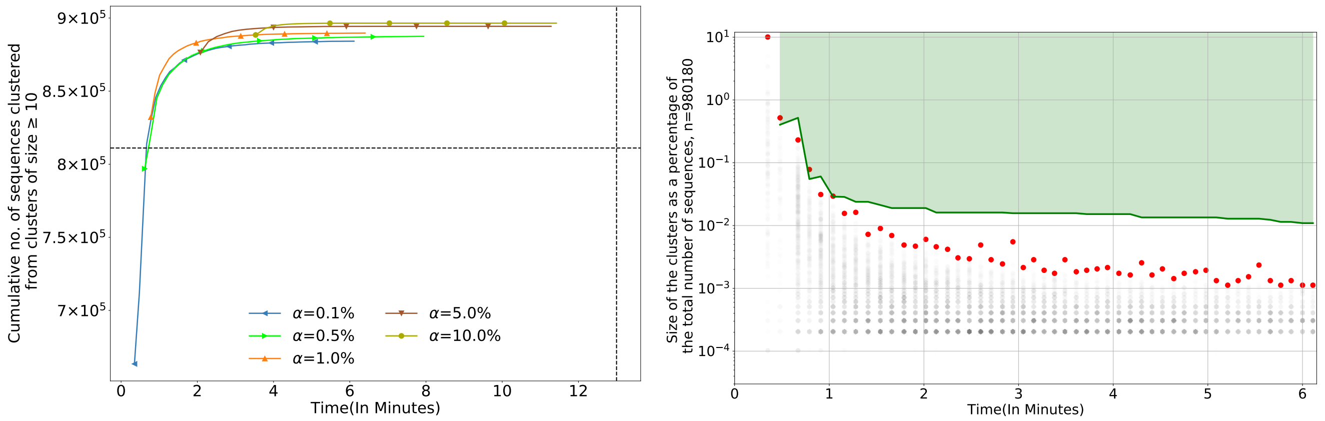

(a) (b)

Figure S3: (a) Time taken per iteration to identify clusters of size at least 10 on the lupus microbiome data set. The dashed lines represent the number of sequences belonging to clusters of size at least 10 and the total time taken by DNACLUST when run stand alone (b) Distribution of cluster sizes on the lupus microbiome data set for the clusters discovered by each round of SCRAPT with time along X-axis. The red dots indicate the size of the largest cluster identified in each iteration. The shaded region represents the credible region identified by the bootstrapping procedure of SCRAPT, specifically an upper bound on the size of clusters that may be detected in that iteration.

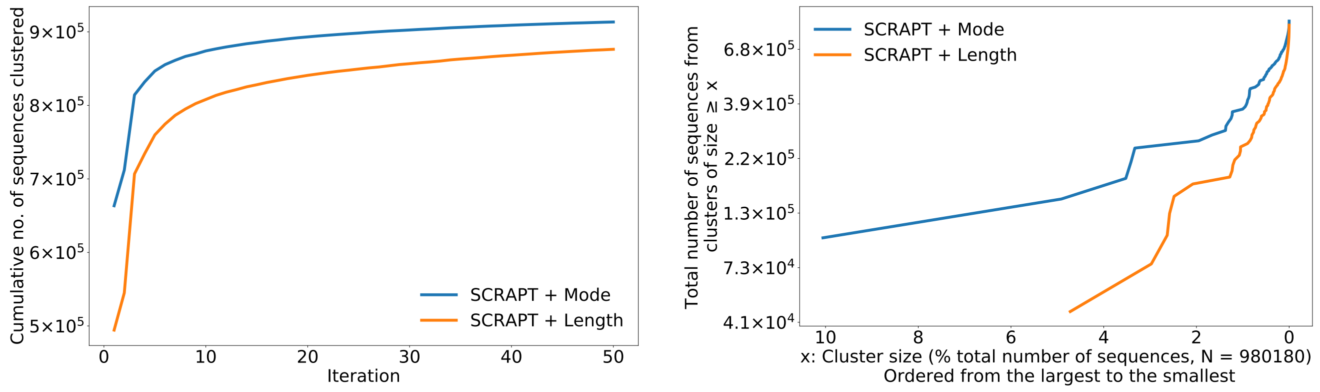

(a) (b)

Figure S4: (a) Cumulative number of sequences clustered by SCRAPT with mode and longest sequence as the choice of centroid. (b) Measure of fragmentation for clusters produced by running SCRAPT with mode and longest sequence as the choice of centroids.

cluster, C, cluster centroids, and E, extended members of the cluster. Extended members can be thought of as weak members that violates the similarity criterion by a small amount. From the figure S5a we see that SCRAPT produces less fragmented clusters compared to MeShClust 3.0 even when its extended members are considered. In figure S5b we compared the run-times between SCRAPT and MeShClust 3.0 and we see that SCRAPT is orders of magnitude faster than MeShClust 3.0.

3.6 Comparing the extent of fragmentation between SCRAPT and DADA2

We computed the extent of fragmentation between DADA2 and SCRAPT. We see from figure S6 that on all three datasets SCRAPT produces less fragmented clusters. However, we see a strong correlation between the sizes of the clusters produced by both these methods. In order to justify, why DADA2 more fragmented clusters, we would need the exact members of the ASVs produced by DADA2.

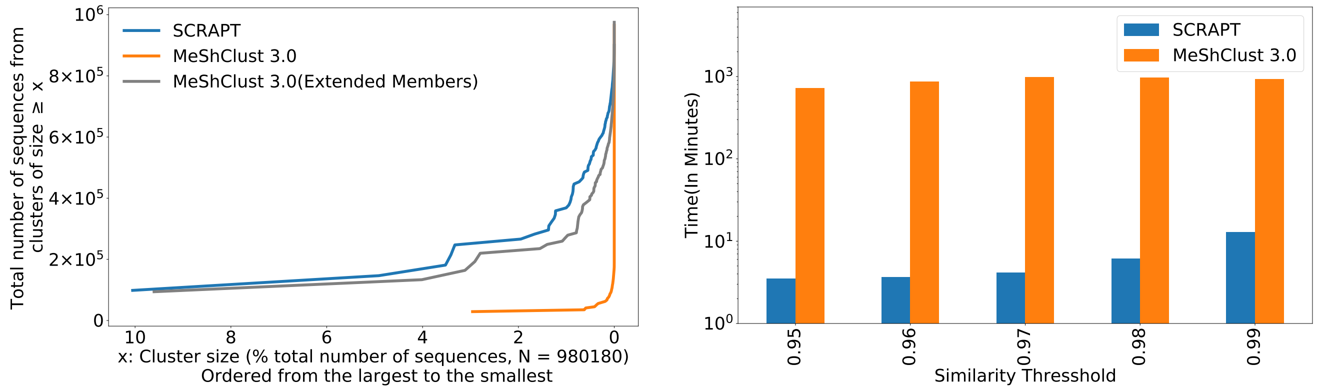

(a) (b)

Figure S5: (a) Fragmentation Measure between `SCRAPT` and `MeShClust 3.0` (b) Comparing Run-times between `SCRAPT` and `MeShClust 3.0` for different similarity thresholds.

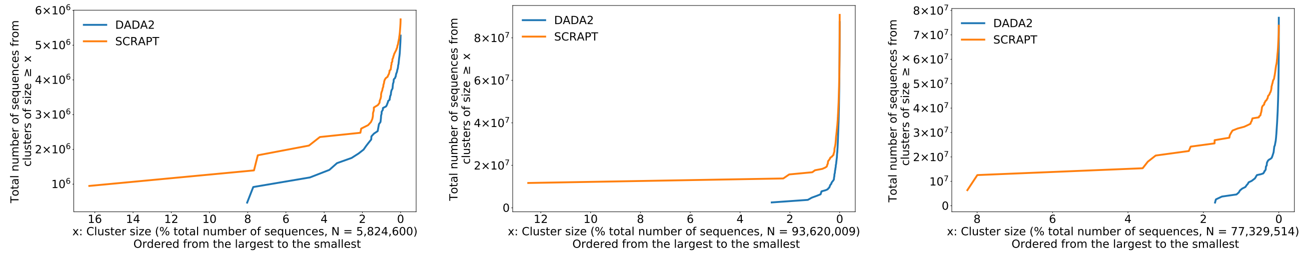

(a) (b) (c)

Figure S6: Fragmentation measure between `SCRAPT` and `DADA2` on (a) Lupus Microbiome Dataset (b) Soil Microbiome Dataset (c) Tara Oceans Dataset.

4 Commands

- We merge the pair-end demultiplexed data using the script multiple_join_paired_ends.py from `qiime2` by the following command

  `multiple_join_paired_ends.py -i [sample directory] -o [joined fastq output directory]`

- We join the demultiplexed merged sequences using the script multiple_split_libraries_fastq.py from `qiime2` by the following command

  `multiple_split_libraries_fastq.py -i [joined fastq directory] -o [output directory] -p [parameters] --demultiplexing_method sampleid_by_file`

- We deduplicated the sequences using the `seqkit rmdup` program.

  `seqkit rmdup -s -D [counts.txt] [sequences.fna] > [deduplicated.fna]`

- The `CD-HIT` experiments were performed using cd-hit "V4.8.1" with the de-duplicated sequence file as the input, with the following command

  `cd-hit-est -g 1 -i [deduplicated.fna] -o [clusters.txt] -c [similarity] -T 8`

- The `UCLUST` experiments were performed using "usearch11.0.667" with the de-duplicated sequence file as the input. It is worth mentioning that `UCLUST` is memory intensive: `UCLUST` on the input data set over 99% similarity fails to finish under 36 gigabytes memory limit, whereas all other tools over the similarities we tested with a maximum memory availability of 36 GB, however `DNACLUST` and `SCRAPT` had a maximum memory usage of 1.6GB.

```
usearch11.0.667_i86linux32 -cluster_fast [deduplicated.fna] -threads 8 -id [similarity]
-fulldp -clusters [output directory]
```

- `DADA2` is a R library and the experiments were performed using "dada2 1.16.0" in R (version "4.1.2") with the original data set as the input. We follow the `DADA2` pipeline given by `https://benjjneb.github.io/dada2/tutorial.html`, with merged pair-end demultiplexed sequences as inputs, for the fairness of comparison

- The `DNACLUST` experiments were run using the `DNACLUST` release 3 and the following commands were used extensively. To run `DNACLUST` to cluster sequences we used,

  ```
  dnaclust [sequences.fa] -s [similarity] -t 8 --no-k-mer-filter > [clusters.txt]
  ```

  To bait sequences using a set of centers, we used the following command.

  ```
  dnaclust [sequences.fna] -s [similarity] -p [centers.fna] -r -t 8 --no-k-mer-filter >
  [clusters.txt]
  ```

- The experiments involving iterative clustering were performed using `SCRAPT`. The following commands were used,

  – Adaptive sampling with mode-shifting: `python SCRAPT.py -f [sequences.fna] -o [output directory] -s [initial sampling rate] -a True -d [adjustment constant] -r [similarity] -n 50 -t 8 -m True`

  – Adaptive sampling without mode-shifting: `python SCRAPT.py -f [sequences.fna] -o [output directory] -s [initial sampling rate] -a True -d [adjustment constant] -r [similarity] -n 50 -t 8 -m False`

  – Naive sampling with mode-shifting: `python SCRAPT.py -f [sequences.fna] -o [output directory] -s [initial sampling rate] -a False -d [adjustment constant] -r [similarity] -n 50 -t 8 -m True`

  – Naive sampling without mode-shifting: `python SCRAPT.py -f [sequences.fna] -o [output directory] -s [initial sampling rate] -a False -d [adjustment constant] -r [similarity] -n 50 -t 8 -m False`

- `MeShClust` experiments were run using `MeShClust v3.0`. `MeShClust v3.0` takes the deduplicated sequences and similarity threshold as inputs. We ran `MeShClust` with 8 threads. To run `MeShClust` we ran,

  ```
  meshclust -d [sequences.fna] -o [clusters.txt] -t [similarity thresholds] -c 8
  ```
